# Prioritization of surgical patients during the COVID-19 pandemic and beyond: A qualitative exploration of patients’ perspectives

**DOI:** 10.1371/journal.pone.0294026

**Published:** 2023-11-08

**Authors:** Anouk M. I. A. van Alphen, Sandra Sülz, Hester F. Lingsma, Robert J. Baatenburg de Jong

**Affiliations:** 1 Department of Otorhinolaryngology, Erasmus University Medical Center, Rotterdam, The Netherlands; 2 Erasmus School of Health Policy & Management, Erasmus University Rotterdam, Rotterdam, The Netherlands; 3 Department of Public Health, Erasmus University Medical Center, Rotterdam, The Netherlands; Calcutta National Medical College, Government of West Bengal, INDIA

## Abstract

**Introduction:**

During the COVID-19 pandemic, prioritizing certain surgical patients became inevitable due to limited surgical capacity. This study aims to identify which factors patients value in priority setting, and to evaluate their perspective on a decision model for surgical prioritization.

**Methods:**

We enacted a qualitative exploratory study and conducted semi-structured interviews with N = 15 patients. Vignettes were used as guidance. The interviews were transcribed and iteratively analyzed using thematic analysis.

**Results:**

We unraveled three themes: 1) general attitude towards surgical prioritization: patients showed understanding for the difficult decisions to be made, but demanded greater transparency and objectivity; 2) patient-related factors that some participants considered should, or should not, influence the prioritization: age, physical functioning, cognitive functioning, behavior, waiting time, impact on survival and quality of life, emotional consequences, and resource usage; and 3) patients’ perspective on a decision model: usage of such a model for prioritization decisions is favorable if the model is simple, uses trustworthy data, and its output is supervised by physicians. The model could also be used as a communication tool to explain prioritization dilemmas to patients.

**Conclusion:**

Support for the various factors and use of a decision model varied among patients. Therefore, it seems unrealistic to immediately incorporate these factors in decision models. Instead, this study calls for more research to identify feasible avenues and seek consensus.

## Introduction

The Coronavirus (COVID-19) pandemic pushed healthcare systems to their limits and exposed their vulnerabilities [1, 2]. Due to the surge of COVID-19 patients, hospitals were forced to use their critical care resources more flexibly. Surgical capacity (e.g., personnel and operating rooms (OR)) was shifted to COVID-19 patients which led to the postponement of operations and a growing surgical backlog [3–5]. Prioritizing certain surgical patients became inevitable, triggering a discussion on which strategies to adopt and how to apply such strategies.

Since the outbreak of the COVID-19 pandemic, there has been ongoing research on criteria and methods for setting surgical priorities. Several models, frameworks, and guidelines have been developed to inform prioritization strategies, and optimize resource usage [6–11]. This also led to the development of a decision model within our hospital. This model quantifies the health loss due to delay of surgery, thereby providing an urgency measure for physicians [7].

Notably, the patients’ perspective was frequently neglected during the development of these COVID-19 response tools. Some understanding of their perspective on prioritization is known, but these findings originate from studies executed before the pandemic commenced, and it is unclear whether these factors have changed [12–15]. Studies which were published during the pandemic only evaluated patients’ perspectives on the ethical theories used to underpin prioritization strategies or addressed non-surgical resources such as intensive care unit (ICU) beds [16–18]. How to utilize these ethical theories in clinical practice in accordance with patients’ perspectives on surgical prioritization remains unclear.

The exclusion of patient inputs during COVID-19 policymaking can be partly justified by the acute impact of the pandemic. However, incorporating their perspective is important for three reasons. First, patients are a major stakeholder in healthcare, and their involvement contributes to high quality and sustainable policymaking [19, 20]. Second, it is reasonable to incorporate their perspective from a societal point of view since they pay for the resources allocated and are also subject to the prioritization policies. Third, it is valuable for healthcare professionals to know whether the current prioritization strategy accords with patients’ perspectives since this will likely contribute to effective communication strategies about the policy adopted.

The first, essential step towards involving patients in surgical priority setting is to gain a deep understanding of their perspectives. As such, this exploratory study had a two-fold aim: first, to identify which factors patients value in priority setting and, second, to assess patients’ perspectives on the use of a decision model. By pursuing these aims, this study contributes to the broader conversation on surgical prioritization during times of scarcity. Conducting this exploratory study is essential as a preliminary step to identify these factors for potential integration into quantitative methods, given the persistent challenges posed by personnel shortages and surgical scarcity in the coming years.

## Materials and methods

Given the knowledge gap surrounding patients’ perspectives since the outbreak of the pandemic on surgical prioritization and the use of decision models, an exploratory qualitative study was conducted. This method was deliberately chosen since it is flexible and open-ended, and therefore appropriate for obtaining a rich and detailed understanding of patients’ perspectives. Ethical approval was obtained from the Medical Research Ethics Committee (reference number: MEC-2021-0679) of Erasmus MC. The study was prepared in accordance with the consolidated criteria for reporting qualitative research (COREQ) (see S1 File) [21].

### Participants

Patients from Erasmus MC, a tertiary hospital in the Netherlands, were recruited through email. The invitation email was sent to the entire patient panel of our hospital, which includes approximately 6,000 patients. Convenience sampling yielded 15 participants. Seven participants were members of the patient council that represents the interest of the patients at Erasmus MC and is a formal advisory body to the executive board of the hospital. Eight participants were recruited from the hospital’s patient panel, which is an informal and easy-to-approach panel. All the participants were informed about the study through an email that contained a brief description of the study’s aim and how the study was to be performed. All the participants were asked to provide written informed consent for participation. None of the approached participants declined to partake in an interview after additional information was presented.

### Interviews

Two vignettes were developed to guide the semi-structured interviews. These vignettes represented two fictive patients who are both awaiting surgery for diseases well known by the general public (i.e., breast cancer and cardiac arrhythmia). These two ‘patients’ did not reflect a real-life prioritization dilemma but did provide an example to start the discussion and elicit participants’ views. The patient descriptions were established after plenary research team discussions with clinicians and researchers. Predefined factors, derived from the literature and known to be important in prioritization dilemmas (e.g., age, type of disease), were provided in these patient descriptions [22–28]. During the interviews, participants were encouraged to propose individual factors they considered relevant and, through this, the list of predefined factors was expanded.

Each participant interview started with a short introduction explaining the surgical backlog due to COVID-19 and the ongoing research on surgical prioritization. An illustration displaying the outcome of a decision model was shown to the participant to illustrate the rationale of the model. Subsequently, the first author showed the two vignettes using screen sharing. Participants were asked to read the vignettes and thereafter provide a motivation as to which of these two patients should receive priority and why. After the discussion on the patient descriptions, the participants were asked about their opinion on the use of a decision model in daily practice. Finally, three general questions about their age, sex, and if they had experience with working in the healthcare sector were posed.

Prior to this, a pilot interview was conducted that led to some practical issues being resolved (i.e., showing the patient vignettes through screen sharing on Zoom). The interview guide was not adjusted based on the pilot interview and a detailed overview of the interview guide is attached in S2 File.

### Data collection and analysis

The interviews were performed between October 2021 and January 2022. Online Zoom meetings were scheduled due to the COVID-19 restrictions at that time. The participant and interviewer were both at home and no other people were present during the interviews. Audio and visual recording were used to collect the data. In addition, the interviewer made field notes throughout the interview to document useful contextual information. The first author (female, medical doctor) conducted all the interviews. At the time of this study, the first author was working as a PhD candidate, using qualitative and quantitative research methods to study surgical prioritization. Each interview took 30 to 60 minutes and were all carried out in Dutch. The audio recordings were transcribed and subsequently analyzed. The transcripts were not returned to the participants.

Thematic analysis was used to explore the transcripts. To ensure validity, the first and second authors independently coded the transcripts following an iterative procedure using NVivo software (version 12 Pro for Windows) [29]. Regular meetings took place to discuss the coding in batches of five interviews. Discrepancies were discussed and resolved and, when appropriate, codes were refined. These codes were further combined into overarching themes. Data saturation was considered achieved when no new codes emerged from additional interview analyses. A description of the final coding tree and overarching themes can be found in S3 File. Codes, themes, and quotes have been translated into English for this manuscript.

To minimize the impact of the researchers’ biases, assumptions, and values on the research process and findings, we relied on an interview guide, thereby minimizing the variation in how questions were asked. Additionally, we coded the transcripts independently to ensure that different perspectives were considered during data interpretation and discussed the codes within our team. We believe that the diverse expertise within the research team (medicine, epidemiology, health operations management) led to a well-thorough data discussion from several perspectives.

## Results

Details of the 15 participants are provided in Table 1.

**Table 1:** Characteristics of the patient sample (n = 15).

	Patient council (n = 7)	Patient panel (n = 8)	Total (n = 15)
Age (years), median [IQR^a^]	65 [58–72]	66 [60–72]	65 [59–72]
Sex (female), %	2 (29%)	6 (75%)	8 (53%)
Healthcare worker with clinical duties (yes), %	1 (14%)	3 (38%)	4 (27%)

^a^ IQR = Interquartile range.

The interviews revealed three themes covering multiple aspects. The themes were categorized as: general attitudes towards surgical prioritization, patient-related factors, and the perspectives on a decision model for surgical prioritization.

Data saturation was reached after the analysis of ten interviews. Further analysis of the third batch of five interviews revealed no new themes or factors. We therefore concluded that it was not necessary to recruit any new participants, such that our final sample included just the 15 original participants.

### General attitude towards surgical prioritization

The first theme that emerged from the interviews was the participants’ general attitude to surgical prioritization. Participants repeatedly stated that prioritization is extremely difficult and that they are relieved that it is not their responsibility. Moreover, they showed empathy for the decision-maker.

“I find this dilemma very difficult, but of course physicians think so too. […] These are inhumane decisions, absolutely inhumane.” (participant 12)

“When I think about what physicians have to go through, I do not know how they manage this? Every time I hear or see things (about surgical prioritization), I could cry. How must that be for physicians?” (participant 9)

Several participants noted that surgical prioritization is highly dependent on the physician involved and that it is a non-transparent process. The outcome of the decision-making could therefore be influenced by subjectivity. Some participants even expressed a feeling of mistrust.

“The decisions are made behind closed doors, and everybody has to trust that this is done fairly and mainly on medical grounds.” (participant 1)

“It depends on how assertive the physician is in ensuring that the patient gets his OR spot.” (participant 7)

### Patient-related factors

Participants elaborated on various patient-related factors concerning surgical prioritization. These can be broadly distinguished in two sub-themes: 1) individual patient characteristics prior to the surgery and 2) consequences after surgery.

#### Individual patient characteristics prior to surgery

The viewpoint here focused on factors which preoperatively characterize an individual patient: age, physical functioning, cognitive functioning, behavior, and waiting time. Participants voiced that these individual characteristics should be used to assess the urgency of surgery for each patient. Physical functioning refers to the general wellbeing and ability of a patient to carry out daily activities without restrictions. In terms of prioritization, patients considered physical functioning to be a predictive factor of surgical outcome. Cognitive functioning was mentioned sporadically and considered secondary to age and physical functioning: it should be used as an additional criterion, not as a stand-alone justification.

“I am someone who tends to look at the age first. […] What also plays a role, is that physicians, if all is well, can estimate the postoperative risks. […] They can determine whether, if they send this patient to the intensive care unit, the patient will become a wreck.” (participant 10)

Behavior was frequently brought up by participants. This entails behavior which is related to lifestyle in general (e.g., smoking, physical (in)activity, diet, and alcohol abuse), and also the COVID-19 vaccination status was mentioned. Participants expressed viewpoints both for and against incorporating behavioral factors in prioritization strategies. Among those who argued for considering behavior, their opinion was conditional on certain aspects. These aspects involved intentional bad behavior, rebelling against policymakers, and self-inflicted diseases linked to behavior. Under these circumstances, behavior should be taken into account, implicitly as a punishment. Other participants believed that behavior should never be judged and therefore never used as a prioritization criterion. In general, participants considered “good or bad behavior” to be a normative judgement and highly dependent on one’s own standards. Rewarding patients for good behavior was not elaborated upon during the interviews.

“Alcohol drinking, smoking, and bad living habits in general: it is a slippery slope if you take these into consideration.” (participant 11)

“The older generation were encouraged to start smoking. This is in contrast with the younger generation. They know everything about the risks and that it is unhealthy so, when they start smoking, it is much more a conscious choice.” (participant 4)

Waiting time should be taken into account during surgical prioritization according to participants. This factor is multidimensional. Participants considered the length of the waiting time, the frequency (how often has this surgery already been postponed), and the impact for the individual patient (both clinical and emotional) as important.

“I realize that someone with severe arterial disease who has had surgery cancelled three times could warrant prioritization, even though it is really difficult to take this into consideration. […] You do not know if a patient will compete again with another patient by next week, or if they will undergo surgery in a few weeks.” (participant 1)

#### Consequences of (not) receiving surgery

During the interviews, participants opinioned that it is not solely the preoperative status of the patient that should be taken into account. Participants thought about the consequences of priority setting, and especially the situation where some surgeries have to be postponed. The impact of postponement on survival and quality of life (QoL) were deemed highly important, and frequently suggested as the leading prioritization criteria.

“I think at some point we should focus on the odds of dying and quality of life, and yes, I still find that difficult. Then I step over some very personal elements, and just look at the reality.” (participant 1)

Other consequences that were elaborated upon are emotional consequences. These can be clustered into consequences for the patient awaiting surgery (e.g., anxiety, worrying) and the impact the delay might have on others (e.g., family, loved ones). Participants argued that, just as the waiting patient, others can experience stress or mental pressure through delay. Further, if the patient has dependents such as children or care recipients, they could also physically suffer due to the lack of proper care. However, incorporating these factors into medical decision-making was not deemed feasible by participants.

“We have to keep it rational, because as soon as you take emotions into account, it will be hopeless. Everybody has a different perspective on this. […] The impact on others is just too broad and vague, which raises the question whether you should take it into consideration at all. The dilemma will become even more complex. Preferably, you should only focus on the patient and not on the social environment.” (participant 7)

While reasoning about the consequences of surgical prioritization, participants addressed a wider, contextual perspective and focused on the resource distribution among patients and usage per patient. Participants argued that putting many resources into just one patient is unfavorable, as this could result in fewer available resources for a larger group of patients. This reasoning shows that patients look beyond the individual patient and do not solely use medical criteria in prioritizing. Resources mentioned included bed capacity, ICU stay, personnel, and OR time.

“If patient A can go straight to the regular recovery or just needs two nurses, and patient B needs six nurses… I think this trade-off should also be considered”. (participant 2)

### Perspectives on a decision model for surgical prioritization

The last theme which emerged during the interviews relates to the participants’ views on the use of a model in daily practice. The decision model developed within our hospital was used as an example during the interviews. In voicing their perspectives, participants mentioned two aspects of such a model: acceptance criteria and model usage.

#### Acceptance criteria

Participants expressed several requirements which should be met for a decision model to be accepted. Foremost, participants were favorable about the use of a decision model during surgical prioritization. Further, the model should be simple, pragmatic, and not too extensive. Participants argued that incorporating too many individual characteristics would make implementation of the model unfeasible. Moreover, the model should use scientific and trustworthy data as input, and should be supervised and well understood by physicians. Physicians should be able to easily interpret the results and subsequently explain these to patients. Other criteria related to equity: the model should use the same standards for similar patients, regardless of their status, and there should be a comparable nationwide approach.

“At some point, you have to take the model on trust, because if you make the model too difficult, you will get nowhere.” (participant 1)

“The model should not be a black box, because that ultimately leads to a feeling of mistrust. […] It is important to me that the model is thoroughly evaluated, and maybe goes through some kind of filter at the end.” (participant 2)

#### Model usage

Participants’ views on model usage were explored. As an example, our model output was presented, which involves a ranking of surgical procedures driven by the expected health loss due to a delay in surgery. According to the participants, there are two ways such model output could be used in daily practice. First, participants indicated that the output would be useful for surgical planning as a prioritization tool. Importantly, they felt this tool should support the clinical decision-maker rather than being the sole driver of prioritization decisions. Participants frequently stated that they prefer the physician to be the final decision-maker. Further, physicians should also be able to deviate from the model outcome based on their own clinical judgement. Physicians’ expertise was highly valued by the participants. Sometimes, participants also expressed that a multidisciplinary prioritization committee consisting of physicians, ethicists, and other healthcare professionals (e.g., nurses) would appeal to them. Patient involvement in such a committee was not considered to have added value by most of the participants.

“It is the physician who is in charge of the decision, I think that should be clear. It is not up to the patient. In my opinion, in all cases, the decision must be made by the physician.” (participant 4)

“I do think that the prioritization decision should not be solely made by the physician, who of course best knows the patient’s story, but together with a committee. This committee should consist of an ethicist and other physicians who are not familiar with the patient.” (participant 1)

“I think that it is very important that the physician also takes his personal knowledge about the patient into consideration during prioritization, together with the model output.” (participant 3)

Second, most of the participants suggested using the model output as communication material during a consultation. An illustration of the output could be helpful and provide useful insights about the ongoing prioritization dilemmas. Some saw disadvantages as this explanation could lead to more distress and result in a very impersonal feeling if the model was used to explain dilemmas on an individual level.

“I think that this model is the way to calm people down. This model provides insight into the dilemmas that physicians struggle with.” (participant 9)

“I think that showing the model outcome could help put things into perspective regarding your place on the waiting list. A lot of people probably have a quite primitive reaction, such as ‘I am in pain, I want to go first’, or do not realize that there are more dimensions than just sick or healthy. […] If I were a physician, I would use the model as information material to explain to people in an easy and simplified way how difficult the prioritization trade-off is.” (participant 2)

“I think that using this model to explain to a patient why he or she has to wait will only add fuel to the fire and worsen the disease process. In my opinion, this decision should not be openly shared with patients.” (participant 7)

## Discussion

The main purpose of this study was to explore patients’ perspectives on surgical prioritization and identify which factors they value in priority setting. Furthermore, the patients’ perspectives on using a decision model during this process was assessed. We found that, while patients understand the complexity and difficulties of prioritization, they criticize the prioritization process itself. They want greater transparency and are concerned about the impact of subjectivity on decision-making. From the patients’ perspective, allocative decisions should be guided, albeit to varying degrees, by patient-related factors such as age, physical functioning, waiting time, expected gain in QoL and survival, and resource usage. Overall, patients support the use of a decision model in daily practice, but want physicians to make the final decision based on their clinical expertise. Patient involvement in the decision-making process was not deemed to be of added value but they would value an explanation of a model if one was being used.

With respect to allocative decisions, several factors were considered relevant to patients. Factors that were clinically oriented (e.g., age, physical functioning, expected gain in QoL and survival) were considered important and this finding supports previous research into the allocation of other scarce resources [22–24, 27, 30, 31]. Notably, behavioral factors were widely discussed in our interviews. It is known that the general public often supports a more blame-oriented approach, suggesting that the public believes that every patient has some degree of responsibility for their disease [26, 32]. This perspective may have been especially emphasized in our study since the interviews took place during a heated debate in the Netherlands on COVID-19 vaccination hesitancy. Similarly, other studies carried out during the pandemic show that Dutch people attach substantial value to behavioral factors and would penalize patients who do not adhere to the COVID-19 measures or are obese [33].

From the results, we conclude that the patients’ views on a decision model are not straightforward. Consistent with earlier evidence, patients prefer physicians to make the final decision [16, 18, 22]. This attitude accords with other studies evaluating patients’ views on other tools that could support prioritization decisions such as algorithms. The majority of patients feel that algorithms should not be used without the involvement of a physician and, in general, patients are ambivalent about such tools [34, 35]. As the application of algorithms in healthcare continues to evolve, ‘algorithm aversion’ has received considerable attention, with people appearing reluctant to let healthcare providers rely on such algorithms [36]. People tend to discount algorithmic decisions, even when they are proven to be superior, and instead favor human decision-makers. Along with individual preferences and the nature of the task to be executed, the “black-box character” of an algorithm (perceived complexity, lack of transparency, and inaccessible to people) has been advanced as an explanation for this attitude [36, 37]. These factors that contribute to ‘algorithm aversion’ could also play a significant role in the acceptance or rejection of decision models. In further development and adaption, this perception of a model being a “black box” needs to be addressed. Providing stakeholders with sufficient and understandable information on the model could possibly resolve some of this aversion.

This study adds to the broader discussion about surgical prioritization and how to operationalize this in times of scarcity. Given the complexity and novelty of this topic, there was a paucity of evidence on which factors patients value. Therefore, conducting this exploratory study was required as an initial step to shed some light on these factors. Subsequently, these can be integrated into quantitative methods to elicit preferences (e.g., discrete choice experiments). Considering the ongoing challenges due to personnel shortages, surgical scarcity will remain a bottleneck in the upcoming years and therefore this line of research is of great significance.

No research is free of limitations, and our findings should be interpreted in light of these. First, the results of this study might be influenced by selection bias. All the participants are members of the patient council or panel, which implicitly indicates that they want to be involved in the day-to-day affairs of the hospital. This could increase their desire for more explanation and communication about the prioritization dilemmas. Furthermore, the participants do not adequately reflect the diversity of the general population as they are all rather health literate. Finally, some of the participants could have heard about the decision model prior to this study since, in recent years, the model has received attention both inside the hospital as well as nationwide. This prior knowledge could have influenced their attitude, either positively or negatively, towards the model. Likewise, by showing them the model and vignettes during the interviews, participants were possibly primed to report factors already mentioned (e.g., survival and QoL). In general, we would like to stress the exploratory nature of our study. We purposively choose this research design to gain a deep understanding of patients’ perspectives. Though, the results obtained in our study do not provide an exhaustive overview of all possible perspectives that might exist but contribute to extend our understanding of patient perspectives

Despite these limitations, our findings have two major implications for clinical practice and research. First, this study provides an extensive overview of factors that patients consider important when reasoning about prioritization. These factors should be discussed with stakeholders (e.g., physicians, model developers, and policymakers) to see whether it is desirable and feasible to incorporate them in a decision model or other prioritization tools. Further studies should also evaluate how other stakeholders value these factors.

The second call for action addresses providing appropriate information to patients. The participants called for information on the prioritization decisions made. Although they do not want to be involved in the process itself, or want to have a formal say, they do want some explanation. Given the algorithm aversion, it would be beneficial for wider acceptance if we “opened the black box” and explained and educated patients on the prioritization decisions and the potential usage of a prioritization tool to guide these processes. However, it is not clear how to one should effect this information provision since some participants also expressed concerns (e.g., impersonal feelings). We would encourage further studies involving patients to develop a full understanding of their needs and preferences.

## Conclusions

This study evaluated patients’ perspectives on surgical prioritization dilemmas and the use of a decision model to guide these decisions. Whilst a comprehensive set of factors was established, it also became evident that the support for the various factors differed among the participants. Therefore, it seems unfeasible to immediately incorporate all these factors in prioritization strategies or decision models. Instead, this study concludes that more research is needed to identify feasible avenues and seek consensus.

## Supporting information

S1 File Completed COREQ checklist. (DOCX)

S2 File Interview guide. (DOCX)

S3 File Coding tree. (DOCX)
