# Supplementary material for: Prioritization of surgical patients during the COVID-19 pandemic and beyond: A qualitative exploration of patients’ perspectives
Source: PLoS One. 2023 Nov 8;18(11):e0294026. doi: 10.1371/journal.pone.0294026 (PMC10631689; doi:10.1371/journal.pone.0294026)
Supplement: S1 File — (DOCX) [file pone.0294026.s001.docx]

Supporting Information 1 – completed COREQ checklist

| **COREQ item** | **Guide question/description** | **Answer** |
| --- | --- | --- |
| 1. Interviewer/facilitator | Which author/s conducted the interview or focus group? | The first author conducted the interviews. |
| 2. Credentials | What were the researcher’s credentials? E.g. PhD, MD | AA: MD  SS: PhD  HL: PhD  RBdJ: PhD, MD |
| 3. Occupation | What was their occupation at the time of the study? | AA: PhD student  SS: Assistant professor  HL: Professor  RBdJ: Professor |
| 4. Gender | Was the researcher male or female? | The interviewer is female. |
| 5. Experience and training | What experience or training did the researcher have? | All authors have undertaken training in qualitative research methodologies and had experience in conducting interviews. |
| 6. Relationship established | Was a relationship established prior to study commencement? | No prior relationship was established between the researchers and participants. |
| 7. Participant knowledge of the interviewer | What did the participants know about the researcher? e.g. personal goals, reasons for doing the research | The participants knew where the interviewer worked and the objectives of her study. |
| 8. Interviewer characteristics | What characteristics were reported about the interviewer/facilitator? e.g. Bias, assumptions, reasons and interests in the research topic | The main objectives of the study were explained by the interviewer at the outset. |
| 9. Methodological orientation and theory | What methodological orientation was stated to underpin the study? e.g. grounded theory, discourse analysis, ethnography, phenomenology, content analysis | Thematic analysis was used to explore the transcripts. |
| 10. Sampling | How were participants selected? e.g. purposive, convenience, consecutive, snowball | Convenience sampling yielded all participants. |
| 11. Method of approach | How were participants approached? E.g. face-to-face, telephone, mail, email | Patients were recruited via email. |
| 12. Sample size | How many participants were in the study? | There were 15 participants in this study. |
| 13. Non-participation | How many people refused to participate or dropped out? Reasons? | None of the approached participants declined the interview after additional information was presented. |
| 14. Setting of data collection | Where was the data collected? e.g. home, clinic, workplace | The participant and interviewer were both at home |
| 15. Presence of non-participants | Was anyone else present besides the participants and researchers? | The participant and interviewer were both at home and no other people were present during the interviews. |
| 16. Description of sample | What are the important characteristics of the sample? e.g. demographic data, date | For a detailed description, please see Table 1 of the manuscript. |
| 17. Interview guide | Were questions, prompts, guides provided by the authors? Was it pilot tested? | An interview guide containing two vignettes was developed. These vignettes were established after plenary research team discussions with clinicians and researchers. Subsequently, a pilot interview was conducted. Based on this pilot interview, some practical issues were resolved (i.e., showing the patient vignettes via screen sharing on Zoom). The interview guide was not adjusted based on the pilot interview. |
| 18. Repeat interviews | Were repeat interviews carried out? If yes, how many? | We only interviewed the participants once. |
| 19. Audio/visual recording | Did the research use audio or visual recording to collect the data? | Audio and visual recording were used to collect the data. |
| 20. Field notes | Were field notes made during and/or after the interview or focus group? | The interviewer made field notes throughout the interview to document useful contextual information. |
| 21. Duration | What was the duration of the interviews or focus group? | Each interview took 30 to 60 minutes in total. |
| 22. Data saturation | Was data saturation discussed? | Data saturation was reached after analysis of 10 interviews. Further analysis of the third and last batch of interviews revealed no new themes or factors. We therefore concluded that no additional recruitment of new participants was necessary, which led to the definitive number of 15 participants in our study. |
| 23. Transcripts returned | Were transcripts returned to participants for comment and/or correction? | The transcripts were not returned to the participants. |
| 24. Number of data coders | How many data coders coded the data? | The first and second author independently coded the transcripts. |
| 25. Description of the coding tree | Did authors provide a description of the coding tree? | A description of the final coding tree and overarching themes can be found in Supporting information file 3. |
| 26. Derivation of themes | Were themes identified in advance or derived from the data? | Pre-defined factors, derived from literature, which are known to be important in prioritization dilemmas (e.g., age, type of disease) were used for the vignettes. During the interviews, participants were encouraged to propose individual factors they considered relevant. Thereby, the pre-defined factors were expanded and refined into overarching themes. |
| 27. Software | What software, if applicable, was used to manage the data? | NVivo software (version 12 Pro for Windows) was used to manage the data. |
| 28. Participant checking | Did participants provide feedback on the findings? | Participants did not provide any feedback on the findings. |
| 29. Quotations presented | Were participant quotations presented to illustrate the themes / findings? Was each quotation identified? e.g. participant number | Quotations have been presented throughout the Results section in the manuscript. Quotations are identified by participant number. |
| 30. Data and findings consistent | Was there consistency between the data presented and the findings? | We endeavoured to report our study findings in a clear and concise manner in the Results section of the manuscript, in order to accurately reflect our collected data. |
| 31. Clarity of major themes | Were major themes clearly presented in the findings? | The major themes are represented in the Results section of the manuscript. |
| 32. Clarity of minor themes | Is there a description of diverse cases or discussion of minor themes? | These sub-themes can also be found in the Results section of the manuscript. |
