# Supplementary material for: Prioritization of surgical patients during the COVID-19 pandemic and beyond: A qualitative exploration of patients’ perspectives
Source: PLoS One. 2023 Nov 8;18(11):e0294026. doi: 10.1371/journal.pone.0294026 (PMC10631689; doi:10.1371/journal.pone.0294026)
Supplement: S2 File — (DOCX) [file pone.0294026.s002.docx]

## Supporting Information 2 - Interview guide

(Informed consent has been signed before the start of the interview)

Explanation of decision model:

- Thank you for agreeing to speak with me about surgical prioritization.

- Brief explanation of semi-structured interview.

- Brief explanation of the decision model. A picture of the urgency ranking, as calculated by the model, will be shown.

- Your input as a patient representative is very useful to gain further insight into this topic. I am very interested in your views, experiences, and insights. There are no right or wrong answers. We would encourage you to share all information, which you might consider relevant, with us.

- We will start with two vignettes, describing two patient cases. I would like to ask you which patient should receive priority and why.

- Any questions before we start?


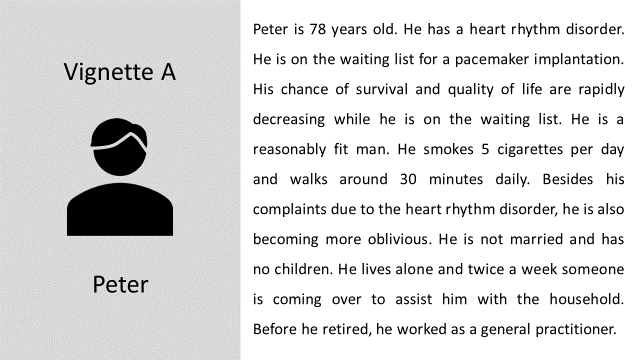


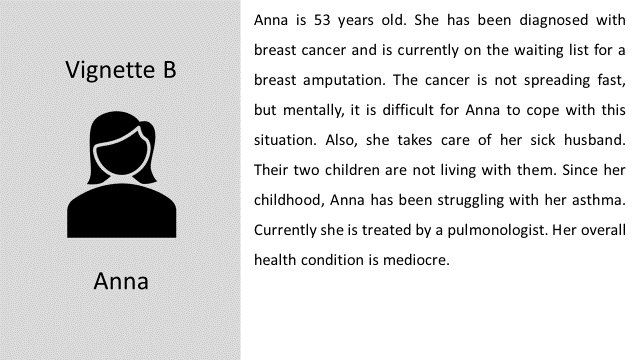


Additional questions:

Who should receive surgery first? And who has to wait?

According to our decision-model, Peter will receive priority. Do you agree?

If the participant agreed: Under which circumstances should Peter receive priority?

If the participant disagreed: Under which circumstances should Anna receive priority?

Would it be acceptable for you if the decision model output would be used? How should the model be used in the process of prioritizing patients for surgery?

Could you come up with an example to illustrate this?

Examples the interviewer can give, to start the conversation:

- Facilitate the discussion between physicians and assist them

- Determine the order of patients (directive approach)

- Inform the physicians during a surgical planning meeting

- Use the ranking as a tool during the consultation

Lastly, I would like to ask you three general questions:

1. What is your age?
2. What is your sex?
3. Do you have any experience with working in the health care sector?
