# Supplementary material for: Prioritization of surgical patients during the COVID-19 pandemic and beyond: A qualitative exploration of patients’ perspectives
Source: PLoS One. 2023 Nov 8;18(11):e0294026. doi: 10.1371/journal.pone.0294026 (PMC10631689; doi:10.1371/journal.pone.0294026)
Supplement: S3 File — (DOCX) [file pone.0294026.s003.docx]

## Supporting Information 3 - coding tree

This figure shows the coding tree for thematic analysis.

Code description:

View on prioritization decision: the general view of participants on the prioritization of surgical patients, e.g., it was considered a difficult choice, participants were happy that they are not responsible for making this decision, and/or they showed understanding for the difficult decision to be made.

View on process: the process of prioritizing patients could be seen as highly dependent on a physician, an inefficient process and/or non-transparent.

Individual factors: these codes describe various individual factors which could possibly affect allocative decisions.

Principles and data requirements: any requirements or comments on the feasibility of our decision model.

Involved people: participants mentioned which people should be involved if the model output would be used.

Output usage: participants were stimulated to think of situations wherein the model output could be used.
